# Carotid Plaque Hemorrhage on Magnetic Resonance Imaging Strongly Predicts Recurrent Ischemia and Stroke

**DOI:** 10.1002/ana.23876

**Published:** 2013-06-04

**Authors:** Akram A Hosseini, Neghal Kandiyil, Shane T S MacSweeney, Nishath Altaf, Dorothee P Auer

**Affiliations:** 1 Division of Radiological and Imaging Sciences, University of NottinghamQueen's Medical Campus, Nottingham, United Kingdom; 2 Department of Vascular and Endovascular Surgery, Nottingham University HospitalQueen's Medical Campus, Nottingham, United Kingdom

## Abstract

**Objective:**

There is a recognized need to improve selection of patients with carotid artery stenosis for carotid endarterectomy (CEA). We assessed the value of magnetic resonance imaging (MRI)-defined carotid plaque hemorrhage (MRIPH) to predict recurrent ipsilateral cerebral ischemic events, and stroke in symptomatic carotid stenosis.

**Methods:**

One hundred seventy-nine symptomatic patients with ≥50% stenosis were prospectively recruited, underwent carotid MRI, and were clinically followed up until CEA, death, or ischemic event. MRIPH was diagnosed if the plaque signal intensity was >150% that of the adjacent muscle. Event-free survival analysis was done using Kaplan–Meier plots and Cox regression models controlling for known vascular risk factors. We also undertook a meta-analysis of reported data on MRIPH and recurrent events.

**Results:**

One hundred fourteen patients (63.7%) showed MRIPH, suffering 92% (57 of 62) of all recurrent ipsilateral events and all but 1 (25 of 26) future strokes. Patients without MRIPH had an estimated annual absolute stroke risk of only 0.6%. Cox multivariate regression analysis proved MRIPH as a strong predictor of recurrent ischemic events (hazard ratio [HR] = 12.0, 95% confidence interval [CI] = 4.8–30.1, *p* < 0.001) and stroke alone (HR = 35.0, 95% CI = 4.7–261.6, *p* = 0.001). Meta-analysis of published data confirmed this association between MRIPH and recurrent cerebral ischemic events in symptomatic carotid artery stenosis (odds ratio = 12.2, 95% CI = 5.5–27.1, *p* < 0.00001).

**Interpretation:**

MRIPH independently and strongly predicts recurrent ipsilateral ischemic events, and stroke alone, in symptomatic ≥50% carotid artery stenosis. The very low stroke risk in patients without MRIPH puts into question current risk–benefit assessment for CEA in this subgroup. ANN NEUROL 2013;73:774–784

The efficacy of carotid endarterectomy (CEA) in secondary prevention of stroke in patients with symptomatic severe carotid artery stenosis is well documented by pooled randomized controlled trial evidence.^1^ Current guidelines recommend early surgical intervention for symptomatic individuals with 50 to 99% carotid stenosis as determined by angiographic or ultrasonographic measurement of the luminal diameter according to the North American Symptomatic Carotid Endarterectomy Trial (NASCET) criteria.^2^ However, 70 to 80% of symptomatic patients with ≥50% stenosis will not experience recurrent stroke at 5 years.^3^,^4^ This group of patients at low risk of recurrent cerebral ischemic events routinely undergo potentially unnecessary surgical intervention, demonstrating the limitation of the current risk stratification model, based on degree of stenosis alone.^5^

Randomized controlled trials were mostly performed more than a decade ago, since which time there has been considerable progress in best medical management for secondary prevention of stroke. Since then, the EXPRESS study has successfully changed clinical practice to early initiation of medical therapy after transient ischemic attack (TIA) or minor stroke, lowering the risk of stroke recurrence.^6^ This improvement in medical treatment may also reduce the additional benefit from surgery. Nonetheless, some recent guidelines recommend expanding the indications for carotid revascularization in carotid disease.^7^ A cost-effective and reliable method of defining stroke risk beyond that predicted by the degree of stenosis alone would offer the potential to better target patients most likely to benefit from surgery, while avoiding unnecessary surgery for those at low risk of embolic stroke from carotid disease.

Detection of ultrasonographic microembolic signals by transcranial Doppler can be used to assess patients at high risk of recurrence,^8^ but has a limited power in accurately identifying those at very low risk who may be safely excluded from carotid intervention. Other research efforts focused on noninvasive imaging techniques to predict the “vulnerable” or “unstable” plaque based on the evidence that certain histomorphological plaque features are associated with symptomatic carotid disease.^9^ Atherosclerotic plaque destabilization is histologically characterized by fibrous cap rupture, high lipid content, and notably, intraplaque hemorrhage.^10^,^11^ Recently developed dedicated magnetic resonance imaging (MRI) techniques allow characterization of these features,^12^ of which intraplaque hemorrhage (PH) is the most widely studied imaging marker. Based on typical MRI characteristics of blood products, PH can be reliably identified in both multicontrast and single T1-weighted MRI scans that depict PH as distinct intraplaque hyperintensity.^13^–^16^

MRI-defined PH (MRIPH) accurately predicts the histologically defined vulnerable plaque (type VI atherosclerotic plaque as defined by the American Heart Association).^15^ Conversely, MRIPH was shown to be associated with previous, acute, or recent ipsilateral cerebral infarcts,^17^–^21^ acute and chronic cerebral ischemic lesion burden,^22^,^23^ and accelerated recurrent ipsilateral cerebral infarction.^24^ It may also indicate accelerated plaque growth.^25^,^26^ The notion that MRIPH may be considered a marker of thromboembolic plaque activity was underpinned by its association with microembolic signals during CEA,^27^ and with spontaneous microembolic signals.^23^ Further support comes from an association of MRIPH with acute diffusion abnormalities, and in particular with multiple diffusion abnormalities of multiple ages indexing recurrent recent embolic events.^23^ Importantly, MRIPH may predict recurrence and incidence of cerebrovascular ischemic events, such as stroke, TIA, or amaurosis fugax (AmF) in both symptomatic and asymptomatic carotid artery stenosis.^19^,^24^,^28^–^34^ It is however still unclear whether MRIPH can predict recurrent stroke alone, which would need to be demonstrated before MRIPH could be used for risk stratification and selection criteria for invasive therapy. Moreover, the reported studies are limited in sample size and observed events, which led to inaccurate estimates of the predictive power of MRIPH.

Before the implementation of fast-track surgical intervention, we undertook several prospective MRIPH studies in patients with significant symptomatic carotid artery disease. Their clinical management followed best clinical practice at the time of recruitment, so that CEA was performed later and possibly less frequently than in today's practice. Based on this pool of data, we were able to build the largest and arguably a unique longitudinal MRIPH study in patients with symptomatic moderate to severe carotid disease (50–99%). The aim of this extended follow-up study was to determine the predictive value of MRIPH for better stroke risk stratification. To achieve this, we assessed (1) whether MRIPH independently predicts ipsilateral stroke, (2) how strongly MRIPH predicts all recurrent ipsilateral ischemic events, and (3) the annual risk of recurrent events in patients without MRIPH. We then performed a systematic review and meta-analysis of published data on the predictive value of MRIPH.

## Patients and Methods

### Study Population

The study population included the pooled data from 3 prospective single-center observational studies undertaken between October 2002 and September 2009 following identical recruitment protocols and procedures as previously described^30^,^31^,^35^ with new extended follow-up. All patients were consecutively identified from TIA clinics or vascular clinics at the Queen's Medical Centre, Nottingham. Initial cerebral ischemic events including hemispheric TIA, AmF, or nondisabling stroke were confirmed by a clinical consultant or fellow with special interest in stroke medicine or vascular surgery. Participants were recruited into the study if they had had an ischemic event within the previous 6 months, no contraindications to MRI, and a life expectancy of >2 years.

Clinical treatment and all clinical follow-up assessments were performed blinded to the results of the carotid MRI.

### Imaging Protocol

As part of clinical care, all participants had undergone carotid Doppler ultrasonography prior to recruitment. Subjects with ≥50% stenosis were recruited, provided their index cerebrovascular ischemic event was ipsilateral to the carotid artery disease. The degree of stenosis was graded by using ultrasound criteria adapted from the angiographic measurements of the North American Symptomatic Carotid Surgery Trial as used in the Carotid and Vertebral Artery Transluminal Angioplasty Study.^36^ If carotid Doppler was unable to estimate reliably the degree of stenosis, magnetic resonance angiography was performed.

At entry to the study, consenting participants were assessed for cardiovascular risk factors and underwent brain MRI, performed on 1 of the following 1.5T scanners: Vision (Siemens Healthcare, Erlangen, Germany), Intera (Philips Medical Systems, Best, the Netherlands), or Signa (General Electric, Milwaukee, WI), using standard receive-only quadrature neck array coils, as described previously.^30^,^31^,^35^ All patients underwent a coronal T1-weighted 3-dimensional gradient echo sequence with effective blood nulling and fat suppression due to selective water excitation (repetition time = 10.3 milliseconds, echo time = 4.0 milliseconds, Flip angle = 15, inversion time = 20 milliseconds, field of view = 350 × 300mm, matrix = 256 × 140, 140 partitions, volume thickness = 120–150mm). The acquisition took <5 minutes.

**FIGURE 1:**
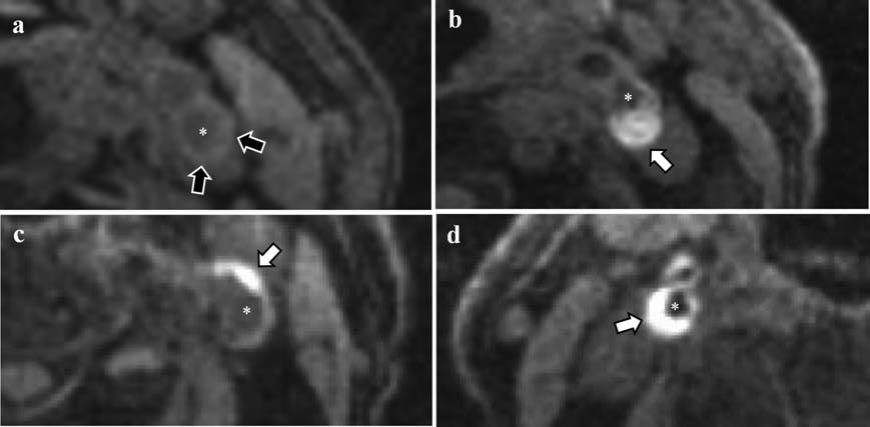
Axial views of T1-weighted water-selective magnetic resonance imaging to detect plaque hemorrhage of carotid arteries. Hyperintense signals (B–D, *white arrows*) reflect plaque hemorrhage in carotid arteries, black arrows (A) show absence of plaque hemorrhage, and asterisks indicate the lumen of internal carotid artery. (A) No signal hyperintensity. (B) Large moderately hyperintense plaque. (C) Small strongly hyperintense plaque. (D) Large strongly hyperintense plaque.

The images were recorded for offline image analysis, which was performed using standard image reconstruction techniques as provided by Jim (Xinapse Systems, http://www.xinapse.com) software. Presence of carotid PH was determined by 2 trained researchers (N.A., N.K.) and adjudicated by an experienced neuroradiologist (D.P.A.), all blinded to the clinical data. Although the presence of PH is readily detectable in the vast majority of cases (Fig 1), the classification used for this study was based on the ratio of the signal intensity within the most hyperintense plaque component relative to that of adjacent sternocleidomastoid muscle; a ratio >1.5 was defined as MRIPH^+^, and ≤1.5 as MRIPH^−^. We have previously shown an excellent interobserver agreement (Cohen κ = 0.80–0.88).^31^

### Clinical Assessment and Follow-up

Clinical assessments for any cerebrovascular ischemic event (stroke, TIA, or AmF), cardiovascular risk factors, and medications were recorded at the time of recruitment. Follow-up until occurrence of ipsilateral ischemic symptom (primary endpoint) or terminating points was performed by the same researcher for each individual study. All recurrent ischemic events were verified by review of clinical details, and all strokes were confirmed as ischemic by neuroimaging.

Coded data from all 3 studies were pooled for new extended follow-up performed by an independent blinded researcher with training experience in neurology/stroke (A.A.H.), which was in part (26 of 62 cerebrovascular events) reported in Kandiyil et al.^35^ Case notes and the hospital Intranet system for central medical records were reviewed to verify clinical index presentation events and any new ipsilateral cerebral ischemic events (TIA, AmF, or stroke) or terminating points until October 2011. In addition, new atrial fibrillation at the time of recurrent event, contralateral stroke, and myocardial infarction were recorded over the entire follow-up period that ranged from 1 day up to 9 years (1–3,344 days).

All patients gave written informed consent for the original studies as approved by the local ethics committee and research and development department, both of which approved the pooled analysis for extended follow-up.

### Statistical Analysis

We investigated whether the presence of MRIPH predicted ipsilateral ischemic stroke, and separately all ischemic events (TIA, AmF, or stroke) by using Kaplan–Meier survival analysis and log-rank test. Ischemic event rates per 100 person-years were calculated for each outcome, and the formula annual risk = 1 − (exp [−event rate × time]) was used to estimate the absolute annual risk. Time to event was further analyzed for stroke and all ipsilateral ischemic events by use of univariate and multivariate Cox regression analysis for MRIPH and established vascular risk factors including age, sex, diabetes mellitus, hypertension, lipid-lowering drugs, antiplatelet therapy, ischemic heart disease, smoking habit, and degree of ipsilateral carotid stenosis, applying a backward conditional model. To assess whether MRIPH is a nonspecific marker of vascular risk rather than a direct marker of the vulnerability of the affected carotid plaque, we also assessed whether MRIPH predicts myocardial infarction or contralateral stroke using a backward conditional Cox regression model adjusted for sex and degree of carotid stenosis.

Lastly, as the chosen carotid MRI does not allow direct differentiation between intraplaque hemorrhage and associated luminal thrombus, we explored the time dependence of MRIPH in relation to the presenting symptom using regression analysis. All tests were performed using SPSS for Windows (version 18.0; SPSS, Chicago, IL); *p* < 0.05 was considered significant.

### Meta-Analysis

We performed a meta-analysis combining the results from our pooled data with those from other studies that reported the relationship between the presence of MRIPH and ipsilateral cerebral ischemic events including both symptomatic and asymptomatic carotid artery disease. PubMed and Embase were searched between January 1990 and April 2012. Search terms were [TIA OR transient isch(a)emic attack OR amaurosis fugax OR stroke OR cerebral isch(a)emi(*) OR cerebral infarct(*)] AND (*)plaque AND carotid(*). Only articles written in English and reporting results in humans were included. Reference lists of selected articles were also searched for relevant references. Case series and individual case reports were excluded. The inclusion criteria took in symptomatic or asymptomatic carotid artery stenosis using MRI carotid plaque imaging techniques in which hyperintense signal reflected vulnerable carotid plaque. Studies using T2-weighted MRI and contrast-enhanced magnetic resonance angiogram were examined and excluded due to lack of histological validation studies and the likelihood of representing lipid-enriched plaque components other than PH. Studies providing data on cerebral ischemic events prior to the plaque imaging were excluded. Two researchers (A.A.H., D.P.A.) independently extracted data from each study. Meta-analysis was performed using RevMan5 software (Cochrane IMS, http://ims.cochrane.org) by use of a random effect model.^37^

## Results

We included 179 subjects with symptomatic carotid artery disease with ≥50% on the standard ultrasound criteria described in Patients and Methods. This included 127 (70.9%) men and 52 (29.1%) women with a mean age of 71.7 years (range = 41–91, interquartile range = 65–79 years). A total of 114 subjects (63.7%) were identified to have MRIPH (MRIPH^+^). MRIPH was absent (MRIPH^−^) in the remaining 65 patients. Demographic characteristics and risk factors in the study population with or without MRIPH are provided in Table 1. Patients with PH were more likely to be male (as previously reported^35^) and non- or ex-smokers and tended to be less affected by ischemic heart disease (see Table 1). Time from presenting symptom did not affect presence of MRIPH (*p* = 0.65).

**TABLE 1:** Demographic Characteristics and Risk Factors in Participants with and without PH on Ipsilateral Carotid MRI at the Time of Recruitment into the Study

Characteristic	MRIPH^+^, n = 114	MRIPH^−^, n = 65	*p*
Age, median yr (interquartile range)	74.9 (66–79)	73.8 (62–78·5)	0.1

Female, No. [%]	25 [21.9]	27 [41.5]	0.001^a^

Diabetes mellitus, No. [%]	14 [12.3]	6 [9.2]	0.53

Hypertension, No. [%]	91 [79.8]	51 [78.5]	0.66

Ischemic heart disease, No. [%]	28 [24.6]	22 [33.8]	0.09

Statin use, No. [%]^b^	88 [77.2]	52 [80]	0.44

Atrial fibrillation, No. [%]	7 [6.1]	7 [10.8]	0.13

Smoking habit, No. [%]			

Smokers	39 [34]	34 [52]	0.04^a^

Nonsmokers	45 [40]	20 [31]	

Ex-smokers^c^	30 [26]	11 [17]	

Antiplatelet or anticoagulant agents used, No. [%]			0.32

Aspirin	73 [64]	31 [47.7]	

Clopidogrel	3 [2.6]	8 [12.3]	

Dual^d^	31 [27.2]	19 [29.2]	

Warfarin	6 [5.3]	4 [6.2]	

None	1 [0.9]	3 [4.6]	

Degree of Stenosis, No. [%]^e^			0.61

50–69%	43 [37.7]	25 [38.5]	

70–99%	71 [62.3]	40 [61.5]	

Type of symptom on presentation, No. [%]			0.72

Stroke	39 [34.2]	24 [36.9]	

TIA	52 [45.6]	26 [40]	

Amaurosis fugax	23 [20.2]	15 [23.1]	

Time between clinic assessment and MRI, median days (interquartile range)	16.5 (2–40.5)	27 (14.5–64)	0.65^f^

Time between presenting symptom and MRI, median days (interquartile range)	36.5 (16.5–81.2)	45 (24–86.5)	

Time from clinical assessment and carotid endarterectomy, median days	34	55	

Total carotid endarterectomies, No. [%]	82 [72]	38 [58]	

Follow-up until terminating point, mean days (interquartile range)^g^	311 (15.5–105)	924 (44.5–1,863)	

Follow-up until any endpoint, mean days (interquartile range)^h^	303 (15–176)	880 (40.5–1,773)	

New atrial fibrillation at the time of recurrent event, No. [%]	0	3 [4.6]	

a Significantly different (*p* < 0.05) between MRIPH^+^ and MRIPH^−^ groups.

b Patients were on regular statin therapy >6 months prior to inclusion into the study.

c Ex-smokers were defined as having stopped smoking for >6 months.

d Aspirin + (dipyridamol or clopidogrel).

e Based on ultrasound criteria described in Patients and Methods.

f Applying binary regression analysis, MRIPH was used as a dependent variable, with time from index symptom to MRI as the covariate.

g Follow-up period from the entry point until the end of the study period, ipsilateral carotid endarterectomy, or death if the patient did not meet the primary endpoint (recurrent event).

h Follow-up until recurrent ischemic event or terminating endpoint.

MRI = magnetic resonance imaging; MRIPH^−^ = absence of hyperintense signal on MRI; MRIPH^+^ = presence of hyperintense signal on MRI; PH = intraplaque hemorrhage; TIA = transient ischemic attack.

We observed 62 recurrent ipsilateral ischemic events during the follow-up; 57 of these occurred in the MRIPH^+^ subgroup (25 ischemic strokes, 23 TIAs, and 9 AmFs), compared with only 5 ischemic events (1 stroke, 2 TIAs, and 2 AmFs) in the MRIPH^−^ group (Table 2). Myocardial infarction or contralateral strokes was seen in 18 patients (12 MRIPH^+^, 6 MRIPH^−^). New atrial fibrillations were noted in 3 patients at the time of recurrent event (all MRIPH^−^).

**TABLE 2:** Analysis of Recurrent Cerebral Ischemic Events in Symptomatic Patients with ≥50% Carotid Artery Stenosis

					Adjusted for Risk Factors^a^	
	Events, No.	PY	Event Rate per 100 PY	Annual Risk	Hazard Ratio (95% CI)	*p*
**Ipsilateral recurrent stroke, TIA or AmF**						

MRIPH^+^	57	94.6	60.2	45.2%	11.95 (4.8–30.1)	<0.001

MRIPH^−^	5	156.7	3.2	3.1%	1.0	

**Ipsilateral recurrent stroke**						

MRIPH^+^	25	94.6	26.4	23.2%	35.0 (4.7–261.6)	0.001

MRIPH^−^	1	156.7	0.64	0.6%	1.0	

a Adjusted for age, sex, degree of carotid stenosis, and known vascular risk factors as described in Patients and Methods.

AmF = amaurosis fugax; CI = confidence interval; MRI = magnetic resonance imaging; MRIPH^−^ = absence of hyperintense signal on MRI; MRIPH^+^ = presence of hyperintense signal on MRI; PY = person years; TIA = transient ischemic attack.

**FIGURE 2:**
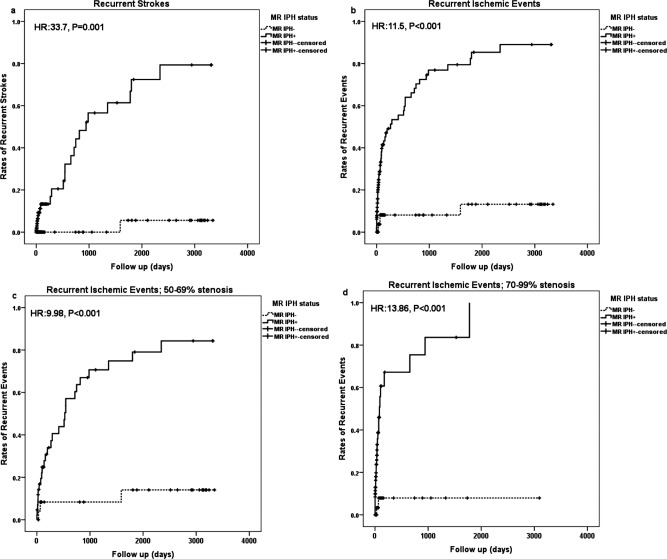
Survival analysis (Kaplan–Meier plot) figures confirm predictive value of magnetic resonance imaging–defined plaque hemorrhage (MRIPH) for (A) stroke and (B) all cerebral ischemic events in both (C) moderate-degree carotid artery stenosis and (D) high-degree stenosis. HR = hazard ratio.

Kaplan–Meier survival analysis demonstrated significantly shorter stroke-free survival for the MRIPH^+^ compared with MRIPH^−^ group (overall chi-square = 28.3, *df* = 1, *p* < 0.001; Fig 2A). Univariate Cox regression analysis for stroke confirmed MRIPH to significantly increase the risk of future ipsilateral ischemic stroke (hazard ratio [HR] = 33.7, 95% confidence interval [CI] = 4.5–251.3, *p* = 0.001). Applying backward conditional modeling, adjusted for known vascular risk factors and time from indexed symptoms to MRI, revealed MRIPH as the only significant factor to predict recurrent stroke (HR = 35.0, 95% CI = 4.7–261.6, *p* = 0.001). Similarly, Kaplan–Meier survival analysis illustrated remarkably different survival curves of participants remaining free of all ipsilateral cerebral ischemic events between those with or without MRIPH (overall chi-square = 41.7, *df* = 1, *p* < 0.001; see Fig 2B).

Univariate Cox regression confirmed MRIPH to notably increase the risk of any future cerebrovascular ischemic events (HR = 11.5, 95% CI = 4.6–28.9, *p* < 0.001). Using backward conditional analysis, only 2 factors showed significant association with recurrent cerebral ischemic events: presence of MRIPH (HR = 12.0, 95% CI = 4.8–30.1, *p* < 0.001) and the degree of stenosis, that is, high-degree (70–99%) stenosis versus moderate-degree (50–69%) stenosis (HR = 1.9, 95% CI = 1.1–3.3, *p* = 0.016).

Applying backward conditional Cox regression showed that MRIPH did not pose a significant risk of contralateral stroke or myocardial infarction during the follow-up period (*p* = 0.95).

Kaplan–Meier analysis of the 2 subgroups classified by luminal narrowing, moderate- versus high-degree stenosis, demonstrated that the presence of MRIPH clearly predicted recurrent ipsilateral events in both moderate-degree (50–69%) and high-degree (70–99%) stenosis (chi-square = 42.1, *df* = 1, *p* < 0.001; see Fig 2C, D).

The estimated risk of any ipsilateral ischemic event at 5 years in ≥50% symptomatic carotid stenosis was significantly higher in MRIPH^+^ compared with MRIPH^−^ patients (85.3%, 95% CI = 74.7–95.9 vs 13.2%, 95% CI = 1.1–25.3, *p* < 0.001). The risk difference between those with and without MRIPH for recurrent stroke was +14.9%, +50.9%, and +66.8% at years 1, 3, and 5, respectively (Table 3).

**TABLE 3:** Risk Estimation for Recurrent Ipsilateral Ischemic Events in Patients with Symptomatic Carotid Artery Stenosis in the Presence of MRIPH

MRIPH^+^ with	Cumulative No. of Patients with Event at 1 Year (at 3 years)	Cumulative Risk^a^ at 1 Year, % [95% CI]	Cumulative Risk^a^ at 3 Years, % [95% CI]	Risk Difference vs MRIPH^−^ Group at 1 Year, %	Risk Difference vs MRIPH^−^ Group at 3 Years, %
≥50% Stenosis	42/114 (53/114)	53.4% [41.1–65.7]	76.9% [65.3–88·5]	+45.3	+68.8

50–69% Stenosis	15/43 (24/43)	40.6%	70.7%	+32.3	+62.4

70–99% Stenosis	27/71 (29/71)	67.2%	83.6%	+59.3	+75.7

a Kaplan–Meier estimate.

CI = confidence interval; MRI = magnetic resonance imaging; MRIPH^−^ = absence of hyperintense signal on MRI; MRIPH^+^ = presence of hyperintense signal on MRI.

For the systematic review and meta-analysis, we identified 3,764 PubMed and 2,771 Embase abstracts. Only 9 papers provided subsequent recurrent ischemic events for their study population. Of these, 2 articles were our own partially published data and were omitted to avoid duplication.^30^,^31^ One paper that met our inclusion criteria did not disclose the relevant raw data,^34^ and was therefore excluded. A total of 6 studies in addition to this study incorporating data from previous publications^30^,^31^ met the criteria for full or partial data extraction for the meta-analysis. Half the studies reported data on symptomatic carotid stenosis,^24^,^28^,^33^ and the remaining 3 had studied asymptomatic carotid stenosis or a combination of both symptomatic and asympotamic.^19^,^29^,^32^

**FIGURE 3:**
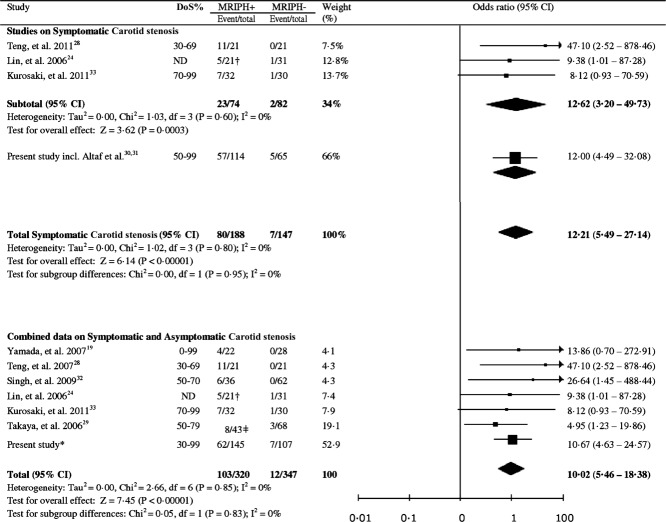
Meta-analysis of available studies on symptomatic carotid arteries (n = 335), and symptomatic combined with asymptomatic carotid arteries (n = 667) to evaluate the association between magnetic resonance imaging (MRI) signal hyperintensity and future risk of ipsilateral cerebral ischemic events. *Combined data including symptomatic carotid artery stenosis and contralateral asymptomatic arteries. †Only included the subgroup of patients with MRI-defined intraplaque hemorrhage (PH) who were followed up for subsequent ischemic events. ‡PH^+^ within lipid-rich necrotic core plaque (LRNC) compared with PH^−^ with LRNC; DoS = Degree of stenosis. CI = confidence interval; ND = not disclosed in the paper.

Meta-analysis on all available data for symptomatic patients (n = 335, 80 events in 188 MRIPH^+^ vs 7 events in 147 MRIPH^−^) confirmed the significant predictive value of MRIPH for ipsilateral cerebral ischemic events (odds ratio [OR] = 12.2, 95% CI = 5.5–27.1; Fig 3).

To control for a potential bias from the effect of the Nottingham data weight of 66%, we repeated the meta-analysis after exclusion of Nottingham data, which showed a similarly strong association that remained significant (OR = 12.6, 95% CI = 3.2–49.7, *p* = 0.0003; heterogeneity: *I*^2^ = 0%, *p* = 0.06).

To further explore whether MRIPH may also be a useful risk marker for future events in asymptomatic carotid disease, we conducted a meta-analysis of all available data for patients with carotid stenosis (see Fig 3). MRIPH was found to be associated with a significantly higher risk of clinical recurrence or new ischemic events in carotid artery disease (OR = 10.0, 95% CI = 5.5–18.4, *p* < 0.00001; heterogeneity: *I*^2^ = 0%, *p* < 0.85). Despite inclusion of patients with asymptomatic disease and differences in MRI technique and degree of stenosis, we did not see significant between-study heterogeneity (*Q* stat = 1.29; *p* = 0.26). The published evidence from studies in patients with asymptomatic carotid stenosis is, however, limited (only a total of 24 events for 282 arteries), and larger cohorts with longer follow-up are needed to confirm the predictive power of MRIPH in asymptomatic carotid stenosis.

## Discussion

We found that MRI-defined carotid plaque hemorrhage independently and strongly predicted recurrent ipsilateral ischemic events and stroke in patients with symptomatic ≥50% carotid artery stenosis. Presence of MRIPH was associated with significantly shorter stroke- and event-free survival using multivariate Cox regression analysis. This translated into an estimated risk difference of 66% for stroke at 5 years.

The strong association of MRIPH and recurrent events makes it a promising biomarker for risk assessment of recurrent events and stroke. We consider plaque hemorrhage as detected by MRI to be a pathophysiologically plausible biomarker of the thromboembolic propensity of carotid plaques. This notion is supported by the relationship between carotid MRIPH and the presence of ipsilateral cerebral ischemic lesion burden,^22^,^23^ microembolic signals during CEA,^27^ and recurrent recent embolic events as detected by multiple white matter diffusion abnormalities as well as spontaneous microembolic signals.^23^ Moreover, 3-dimensional, T1-weighted, blood- and fat-nulled MRI has been histologically qualified to accurately detect plaque hemorrhage in our sample^16^ and by others,^15^ with sensitivity of 91 to 100% and specificity of 77 to 80%, meeting some key biomarker requirements.

Like other biomarkers, including microembolic signals detected by using transcranial Doppler imaging, no direct causative link can be claimed for any given index cerebral ischemic event. MRIPH may simply be an index of overall cardiovascular and stroke risk.^38^ Nevertheless, in our study population the presence of MRIPH in symptomatic carotid artery plaques did not increase the risk of stroke in the contralateral hemisphere or myocardial infarction, adding evidence for a direct link. Arguably, demonstration of previous plaque rupture is a risk factor for future ruptures. This does not exclude the possibility that in some patients, MRI signal hyperintensity may reflect a more recent intraluminal thrombus that may directly cause significantly elevated risk for thromboembolic stroke. The MRI protocol with a 5-minute volume scan that was used did not allow differentiation between plaque hemorrhage and intraluminal thrombus, a limitation that is shared by some histological carotid studies.^38^ In our patient cohort, however, time from the index ischemic symptom to MRI was relatively long (mean = 57 ± 47 days), which reduced the plausibility that the MRI hyperintense signal reflected fresh luminal thrombus. Furthermore, the time from indexed symptoms to MRI did not affect the presence of MRIPH, making intraluminal thrombus an unlikely contribution to the observed MRI hyperintensity.

This interpretation of MRIPH as a cumulative vulnerability marker would explain that the differential risk prediction was not limited to the immediate time period after the initial event, but preserved or even increased for at least 5 years. Further support for the assumption that PH is a relatively stable vulnerability marker of stroke risk comes from longitudinal studies showing substantially stable features of MRIPH.^26^,^39^ Although this makes a direct temporal link with recent plaque rupture unlikely, the stability of PH and its prolonged predictive power for future events are clinically valuable properties, especially for assessment of patients attending clinics outside of the 2-week window that is the ideal time for intervention.

MRIPH was present in 63.7% of symptomatic carotid arteries in our study population of moderate and severe carotid stenosis. This differs from other in vivo plaque studies reporting 28% and 36.7% in 50 to 79% and 50 to 70% asymptomatic carotid artery stenosis, respectively.^32^,^40^ Our reported prevalence of MRIPH is, however, in very close agreement with the 64% PH-positive findings from the Oxford Plaque Study,^41^ a large carotid PH examination of CEA specimens from symptomatic carotid arteries with ≥70% stenosis according to the European Carotid Surgery Trial criteria, hence the degree of luminal narrowing would be estimated at ≥50% stenosis if the NASCET criteria were applied.^42^ The high concordance with histological findings in a patient population similar to ours underlines the accuracy of this imaging technique. Discrepancies in reported prevalence from other in vivo MRI studies might be explained by differences in the study population or limited sensitivity of the techniques. A higher sensitivity of gradient echo over spin echo MRI for detection of PH has been previously demonstrated.^43^ PH as defined by MRI methods has been identified across a wide range of carotid stenosis,^44^ and appears to be a feature of plaque vulnerability across all degrees of luminal narrowing,^19^,^24^,^28^–^33^ even in low-grade stenosis.^34^,^45^

To our knowledge, this is the largest longitudinal MRI study of symptomatic carotid artery disease for prediction of secondary events in vulnerable patients with a follow-up of up to 9 years. The CIs around our estimate rates of recurrent events were still wide, limited by the number of observed recurrent events. However, the 95% CI for the HR of recurrent cerebral ischemic events in our MRIPH^+^ group, adjusted for known vascular risk factors, suggests a minimum HR of 4.7. Notably, this minimum HR carries a higher predictive value for recurrence than other identified clinical risk factors reported from analysis of the results from the NASCET trial. These clinical risk factors include age, sex, stroke being the primary index event, and time period of <2 weeks from the index event, all with HRs of <2.3.^46^ The predictive power of MRIPH also compares favorably with other risk markers, namely cerebral microembolic signals as detected by transcranial Doppler ultrasound, with a reported lower bound of OR of 2 (OR = 4.7, 95% CI = 2.0–11.0, *p* < 0.0001) for prediction of recurrent stroke and TIA in ≥50% symptomatic carotid artery stenosis.^47^ The MRI technique in our study was likewise superior to another assessment tool, ultrasonographic grading of carotid stenosis, with an HR of 12 for MRIPH versus 1.9 for tighter stenosis.

MRIPH has significant potential for improved patient selection, reduction of unnecessary treatment risk, and cost-effective targeted intervention. Notably, we report a cumulative observation period of 156.7 person-years in patients without plaque hemorrhage on best medical treatment only, that is, prior to CEA or study end, during which only 1 stroke occurred (see Table 2). The nominal annual absolute stroke risk of 0.6% is unlikely to outweigh the risk of endarterectomy in many surgical centers. On average, the postprocedural risk of stroke or death within 30 days of CEA or stenting is considered to be between 2.6 and 4.8%,^48^ with even lower rates for specialized centers; a post-CEA risk of 1.1% was recently reported for our regional hospital services.^49^ Although we cannot exclude the possibility that patients without MRIPH may also have a lower perioperative risk of CEA, surgical intervention in the very low-risk MRIPH negative group (0.6% annual risk) might still pose an unacceptably high risk for this subgroup of patients.

Oral medical treatments and CEA were offered to all our study population for secondary prevention according to national and local standard guidelines at the time, and none of the treatments was altered or delayed for the purpose of the studies.

The remarkably low risk of patients with absent MRIPH provides a sound rationale for challenging the risk–benefit assessment for carotid interventions in MRIPH^−^ patients with symptomatic carotid artery stenosis. This is particularly true in subgroups already known to be at lower risk of stroke, such as those presenting >2 weeks from the initial event, female patients, and those with a moderate degree of stenosis and/or high surgical risk. The observed low risk of recurrent or first ipsilateral ischemic events in the absence of MRIPH was not limited to our study population, as confirmed by meta-analysis. A large-scale randomized controlled trial is, however, needed prior to implementation of this biomarker in clinical practice.

The study is limited by a natural history study design with the follow-up periods defined by scheduled CEA for those patients who in accordance with concurrent best clinical care in the United Kingdom at the time of recruiting patients were offered CEA. A large proportion of our patients (67%) underwent CEA with both the decision and timing of CEA based on independent clinical decision making. The current treatment practice in our and other UK centers has recently changed to offering CEA to most patients with at least a 50% degree of stenosis. This makes our reported data set unique and enabled us to demonstrate the strong predictive value of MRIPH status for recurrent events. Importantly, MRIPH status did not affect treatment decisions, and hence we can rule out pertinent bias. Hence, patients with high-degree stenosis were offered CEA and followed up only until scheduled CEA, which led to shorter follow-up and higher rate of censoring in high-grade stenosis. We acknowledge that this may have reduced the predictive power of the degree of stenosis in our multivariate Cox model. Based on recruitment and local audit numbers, we estimate that about 86% of the patient population of our vascular clinics were recruited, which we consider a strength of the study, suggesting that our findings can be considered representative of a tertiary care TIA/stroke service population.

In summary, MRIPH is a strong and independent predictor of risk of recurrent events in symptomatic carotid disease, which may help to assist patient selection for carotid intervention in clinical scenarios with a reduced risk–benefit ratio. The particularly low risk of recurrent events in patients without MRIPH challenges the current risk–benefit assessment for carotid intervention and calls for a randomized controlled trial to assess the benefit of intervention over best medical therapy for patients with low- to intermediate-risk carotid disease, stratified for presence or absence of plaque hemorrhage.
